# Data on association of mitochondrial heteroplasmy and cardiovascular risk factors: Comparison of samples from Russian and Mexican populations

**DOI:** 10.1016/j.dib.2018.02.068

**Published:** 2018-03-12

**Authors:** Tatiana V. Kirichenko, Igor A. Sobenin, Zukhra B. Khasanova, Varvara A. Orekhova, Alexandra A. Melnichenko, Natalya A. Demakova, Andrey V. Grechko, Alexander N. Orekhov, Jorge L. Ble Castillo, Tatiana P. Shkurat

**Affiliations:** a National Research Medical Center of Cardiology, Moscow, Russia; b Southern Federal University, Rostov-on-Don, Russia; c Federal Scientific Clinical Center for Resuscitation and Rehabilitation, Moscow, Russia; d Institute of General Pathology and Pathophysiology, Moscow, Russia; e Institute for Atherosclerosis Research, Skolkovo Innovation Center, Moscow Region, Russia; f Autonomous University Juarez of Tabasco, Villahermosa, Tabasco, Mexico

**Keywords:** CVD, cardiovascular disease, BMI, body mass index, SBP, systolic blood pressure, DBP, diastolic blood pressure, MI, myocardial infarction, HDL, high-density lipoproteins, LDL, low-density lipoproteins, TG, triglycerides, Mitochondrial mutations, Heteroplasmy, Atherosclerosis, Cardiovascular risk factors, Coronary heart disease

## Abstract

Despite the fact that the role of mitochondrial genome mutations in a number of human diseases is widely studied, the effect of mitochondrial heteroplasmy in the development of cardiovascular disease has not been adequately investigated. In this study, we compared the heteroplasmy levels of mtDNA from leukocytes for m.3256C>T, m.3336T>C, m.12315G>A, m.5178C>A, m.13513G>A, m.14459G>A, m.14846G>A, m.15059G>A, m.652insG and m.1555A>G mutations in CVD-free subjects and CVD patients in samples derived from Russian and Mexican populations. It was demonstrated that heteroplasmy level of m.5178C>A was associated with CVD in Russian men, and m.14459G>A – in Russian women. Mitochondrial heteroplasmy level of m.13513G>A and m.652insG were associated with CVD in Mexican men, and only m.652insG– in Mexican women. The levels of heteroplasmy for mitochondrial mutations m.3336T>C, m.5178C>A, m.14459G>A, m.14846G>A and m.1555A>G were significantly higher in CVD-free Mexican men, and for m.3256C>T, m.3336T>C, and m.14459G>A – in CVD-free Mexican women.

**Specifications Table**

**Table:** 

Subject area	Cardiovascular diseases
More specific subject area	Genetic predisposition to cardiovascular disease
Type of data	Tables
How data was acquired	Pyrosequencing, clinical data, biochemical analysis
Data format	Analysed
Experimental factors	Not applicable
Experimental features	Mitochondrial mutations m.1555A>G, m.3256C>T, m.3336T>C, m.5178C>A, m.12315G>A, m.13513G>A, m.14459G>A, m.14846G>A, m.15059G>A, m.652insG were determined using pyrosequencing technology, and their association with CVD was analysed
Data source location	Rostov-on-Don, Russia
	Moscow, Russia
	Villahermosa, Mexico
Data accessibility	Data are provided in this article

**Value of the data**

• The study shows that in genetically and clinically diverse populations, Russian and Mexican ones, the mutations of the mitochondrial genome are differently related to cardiovascular disease.
• In samples from Russian population, mitochondrial heteroplasmy level of m.5178C>A and m.14459G>A were significantly higher in men and women with CVD, respectively. In samples from Mexican population, heteroplasmy level of these mutations was significantly higher in CVD-free study participants. More, in Mexican population, heteroplasmy levels of m.13513G>A and m.652insG were associated with CVD in males, and m.652insG– in females. Higher level of heteroplasmy of mutations m.3336T>C, m.5178C>A, m.14459G>A, m.14846G>A and m.1555A>G was demonstrated in healthy men, and that of m.3256C>T, m.3336T>C, and m.14459G>A – in healthy women.
• Estimation of the associations of as much as possible mitochondrial mutations with risk factors and clinical signs of coronary heart disease and atherosclerosis provides an important source for further investigation of the role of mitochondrial heteroplasmy level in the development of cardiovascular pathology.

## 1 Data

Clinical and laboratory characteristics of Russian and Mexican study participants are presented in Table 1, Table 2.

**Table 1:** Characteristic of Russian study participants.

Variable	Men			Women		
	Healthy	CVD	*p*	Healthy	CVD	*p*
Age, years	62.8(10.5)	61.6(9.0)	.630	62.2(8.5)	62.5(7.6)	.926
BMI, kg/m^2^	26.3(4.0)	27.6(4.3)	.278	26.1(5.9)	27.1(6.3)	.656
SBP, mm Hg	142(18)	138(14)	.358	133(15)	128(11)	.324
DBP, mm Hg	84(12)	82(11)	.499	82(9)	77(7)	.258
Smoking, %	11	15	.684	4	9	.108
Diabetes, %	9	15	.514	7	9	.581
MI, %	0	5	.791	0	0	.853
Total cholesterol, mg/dL	233.9(47.9)	227.8(50.2)	.642	254.3(51.4)	257.5(51.3)	.863
HDL, mg/dL	61.6(15.7)	57.2(12.8)	.271	71.6(14.9)	75.7(14.1)	.438
LDL, mg/dL	147.1(41.6)	138.7(48.7)	.480	160.8(46.1)	153.9(47.2)	.677
TG, mg/dL	125.9(55.5)	159.5(102.7)	.092	109.1(51.1)	139.3(50.8)	.105

Mean (SD) values are shown.

**Table 2:** Characteristic of Mexican study participants.

Variable	Men			Women		
	Health	CVD	*p*	Health	CVD	*p*
Age, years	58.6(11.3)	61.5(10.4)	.219	60.5(10.1)	63.8(8.4)	.217
BMI, kg/m^2^	30.0(4.9)	28.5(6.5)	.242	30.8(4.6)	31.0(6.7)	.900
SBP, mm Hg	127(21)	124(16)	.394	136(22)	135(20)	.888
DBP, mm Hg	78(11)	75(9)	.137	77(8)	73(9)	.137
Smoking, %	44	56	.281	20	38	.160
Diabetes, %	19	51	.002*	30	50	.144
MI, %	0	35	<.001*	0	21	.008*
Total cholesterol, mg/dL	208.2(44.5)	159.4(49.7)	<.001*	209.1(50.0)	178.7(51.2)	.037*
HDL, mg/dL	42.0(8.1)	34.0(10.8)	<.001*	8.1(8.8)	39.7(10.9)	.004*
LDL, mg/dL	123.8(33.8)	85.9(36.4)	<.001*	116.4(33.6)	104.5(40.1)	.263
TG, mg/dL	212.1(102.0)	193.4(66.6)	.563	223.6(141.6)	169.1(93.6)	.122

Mean (SD) values are shown.

* Statistically significant difference at *p*<.05.

Table 3 demonstrates statistical significance of the differences in clinical and biochemical characteristics between Russian and Mexican study participants.

**Table 3:** Comparison of Russian and Mexican populations.

Variable	Men		Women	
	Healthy, *p*	CVD, *p*	Healthy, *p*	CVD, *p*
Age, years	.093	.955	.508	.659
BMI, kg/m^2^	.001*	.569	.002*	.118
SBP, mm Hg	.002*	.001*	.657	.319
DBP, mm Нg	.021*	.005*	.055	.256
Smoking, %	.001*	<.001*	.056	.089
Diabetes, %	.210	.001*	.026*	.020*
MI, %		.009*		.022*
Total cholesterol, mg/dL	.019*	<.001*	.001*	<.001*
HDL, mg/dL	<.001*	<.001*	<.001*	<.001*
LDL, mg/dL	.011*	<.001*	<.001*	.009*
TG, mg/dL	<.001*	.396	<.001*	.241

* Statistically significant difference at *p*<.05.

Mitochondrial heteroplasmy level in Russian and Mexican study participants is presented in Table 4, Table 5.

**Table 4:** Mitochondrial heteroplasmy level of Russian participants.

Mitochondrial heteroplasmy, %	Men			Women		
	Healthy	CVD	*p*	Healthy	CVD	*p*
m.12315G>A	27.8(22.9)	26.6(16.1)	.803	32.4(15.7)	34.0(15.0)	.777
m.3256C>T	21.2(16.8)	18.9(10.2)	.561	22.2(12.3)	23.5(14.8)	.770
m.3336T>C	7.9(5.6)	10.5(21.3)	.452	7.8(7.9)	8.5(3.5)	.762
m.5178C>A	10.5(12.1)	17.9(15.9)	.044*	16.0(4.2)	18.3(5.8)	.185
m.13513G>A	27.8(24.6)	31.0(21.4)	.619	23.8(13.2)	21.4(13.1)	.609
m.14459G>A	38.5(26.7)	31.9(26.9)	.362	18.5(9.2)	28.7(16.9)	.019*
m.14846G>A	14.1(17.1)	15.7(17.8)	.731	16.5(19.8)	13.1(4.8)	.575
m.15059G>A	35.2(32.5)	23.1(14.0)	.636	36.3(13.1)	43.7(11.0)	.106
m.652insG	28.6(22.5)	26.8(19.3)	.752	15.7(17.2)	15.6(16.5)	.994
m.1555A>G	17.4(16.4)	17.2(9.5)	.954	16.7(8.9)	18.3(10.1)	.637

Mean (SD) values are shown.

* Statistically significant difference at *p*<.05.

**Table 5:** Mitochondrial heteroplasmy level of Mexican participants.

Mitochondrial heteroplasmy, %	Men			Women		
	Healthy	CVD	*p*	Healthy	CVD	*p*
m.12315G>A	5.4(8.5)	4.0(3.4)	.260	2.7(2.6)	2.7(1.8)	.998
m.3256C>T	10.5(1.9)	10.1(2.1)	.364	10.4(1.9)	9.4(1.7)	.043*
m.3336T>C	2.6(0.8)	1.7(0.5)	<.001*	2.6(9.6)	1.6(5.6)	<.001*
m.5178C>A	10.3(27.7)	1.7(0.8)	.017*	1.6(6.8)	7.6(20.7)	.122
m.13513G>A	18.3(7.2)	27.8(9.8)	<.001*	20.0(6.6)	23.2(7.2)	.098
m.14459G>A	3.1(0.2)	2.8(0.2)	<.001*	3.2(2.5)	2.7(0.2)	<.001*
m.14846G>A	11.5(4.9)	10.0(1.8)	.034*	10.9(2.7)	9.8(2.2)	.106
m.15059G>A	3.1(0.6)	3.6(1.5)	.085	3.1(0.6)	3.4(0.7)	.216
m.652insG	20.0(7.1)	26.0(17.6)	<.001*	21.1(7.3)	27.5(7.4)	.003*
m.1555A>G	30.0(43.6)	12.1(5.8)	.002*	15.7(7.9)	21.8(29.2)	.285

Mean (SD) values are shown.

* Statistically significant difference at *p*<.05.

In samples from Russian population, heteroplasmy level of m.5178C>A is significantly higher in male study participants with CVD than in healthy men; heteroplasmy level of m.14459G>A prevails significantly in women with CVD.

In the sample from Mexican population, heteroplasmy level of m.13513G>A and m.652insG prevails significantly in men with CVD, heteroplasmy level of m.3336T>C, m.5178C>A, m.14459G>A, m.14846G>A and m.1555A>G are significantly higher in healthy men; m.652insG is significantly higher in female study participants with CVD, and m.3256C>T, m.3336T>C, m.14459G>A – in CVD-free women.

Table 6 demonstrates statistical significance of the difference of mitochondrial heteroplasmy level between Russian and Mexican study participants.

**Table 6:** Comparison of mitochondrial heteroplasmy level in Russian and Mexican study participants.

Mitochondrial heteroplasmy	Men		Women	
	Health, *p*	CVD, *p*	Health, *p*	CVD, *p*
m.12315G>A	<.001*	<.001*	<.001*	<.001*
m.3256C>T	.001*	<.001*	<.001*	<.001*
m.3336T>C	<.001*	.002*	.001*	.010*
m.5178C>A	.957	<.001*	<.001*	.030*
m.13513G>A	.037*	.355	.172	.598
m.14459G>A	<.001*	<.001*	<.001*	<.001*
m.14846G>A	.416	.015*	.137	.009*
m.15059G>A	<.001*	<.001*	<.001*	<.001*
m.652insG	.040*	.803	.122	.006*
m.1555A>G	.081	.006*	.647	.701

* Statistically significant difference at *p*<.05.

## 2 Experimental design, materials and methods

Previously we have developed a quantitative assay of mutant allele measurement for mitochondrial heteroplasmic mutations [1] and demonstrated significant differences between unaffected areas and atherosclerotic lesions in human aortic intima [2]. Further the association of mitochondrial genetic variation with vascular diseases and carotid atherosclerosis has been demonstrated [3], [4], [5], [6].

In this study, the association of heteroplasmy level of mitochondrial mutations with CVD in Russian and Mexican populations was estimated. In total, 300 participants (150 in Russia, and 150 in Mexica) were included in the study. Men and women aged from 55 to 79 years (for women – at least five years after menopause). Study participants were divided into CVD-free and CVD group by the results of cardiological examination. CVD group included patients who have been observed by a cardiologist with diagnosed CVD.

The observed levels of heteroplasmy did not reach the necessary level for the development of mitochondrial disorders in this study, since it is known that the level of mitochondrial heteroplasmy in patients should exceed 50% to evolve clinical manifestations [7].

The study was conducted in accordance with the Helsinki Declaration of 1975 as revised in 1983; all participants gave their written informed consent prior to their inclusion in the study.

Mitochondrial DNA was isolated by phenol-chloroform extraction [8]. Polymerase chain reaction (PCR) was used in order to obtain DNA fragments containing the region of the investigated mutations [1]. Analysis of the heteroplasmy level was carried out in the investigated mutations using the original quantitative method previously developed on the basis of pyrosequencing technology [9]. The level of heteroplasmy, i.e. % mutant copies of mtDNA from their total amount in the sample was estimated.

Statistical analysis was performed using the IBM SPSS 20.0 software (IBM Inc., USA). Data are expressed in terms of means and standard deviation.
